# Diseases of Despair: A Commentary

**DOI:** 10.13023/jah.0302.01

**Published:** 2021-05-03

**Authors:** Tim Thomas

**Affiliations:** Appalachian Regional Commission

**Keywords:** Appalachia, diseases of despair, health inequities, health disparities

## Abstract

Across the nation, and within Appalachia, communities that struggle economically experience greater health challenges, with disparities observed across leading causes of death. Within our region, these disparities are particularly notable across diseases of despair.

The Appalachian Regional Commission (ARC) is a unique partnership between the federal government and the 13 Appalachian states, focused on building community capacity and strengthening economic growth in the 420 counties across the Appalachian Region. To fulfill this mission, ARC considers those factors that are necessary for robust economic development. Among those factors is the health of our people.

Across the nation, and within Appalachia, communities that struggle economically experience greater health challenges, with disparities observed across leading causes of death. Within our region, these disparities are particularly notable across diseases of despair, as highlighted in this issue.^1^ It is important to recognize, however, that while poor health outcomes may be a result of economic challenge, at the community level, poor health outcomes may also be a barrier to economic investment. A key finding from the diseases of despair study is that the group aged 25–54 years is most impacted by these causes of death: overdose, suicide, and alcoholic liver disease. These are individuals in their prime working years, and their engagement in our workforce is critical for their own well-being in that it provides them with purpose. That is why we have been focused most recently on helping communities build recovery ecosystems throughout the Region, to give individuals in recovery the services and training they need to ultimately obtain sustainable employment.

Progress toward a healthier Appalachia will further strengthen efforts to make the Region more economically diverse and resilient. The economic challenges of our nation, and of our region, have been exacerbated over this past year due to the COVID-19 pandemic. Additional stressors to our regional economy, treatment systems, and residents are likely to result in increased mortality across diseases of despair. We must all work together to ensure that these challenges are only temporary. This is the time to double down on our commitment to improved health outcomes by leveraging the best of our Appalachian culture—grit, determination, hard work, and compassion.
